# Psychotraumatology in Greece

**DOI:** 10.1080/20008198.2017.1351757

**Published:** 2017-09-29

**Authors:** Gerasimos Kolaitis, Miranda Olff

**Affiliations:** ^a^ Department of Child Psychiatry, Medical School, National and Kapodistrian University of Athens, Aghia Sophia Children’s Hospital, Athens, Greece; ^b^ Department of Psychiatry, Academic Medical Center, University of Amsterdam, Arq Psychotrauma Expert Group, Diemen, The Netherlands

Psychological trauma is very common, understudied and consequently undertreated in Greece and many other countries. The word trauma comes from the Greek trauma (τραύμα) meaning trauma wound, alteration of trōma; akin to Greek titrōskein = to wound, tetrainein = to pierce. Although there is no data available on the prevalence rates of trauma or posttraumatic stress disorder (PTSD) in Greece, there is no reason to believe that various types of traumatic experiences are not common phenomena in Greek society.

Greece, often called the cradle of Western civilization, historically known as Hellas, is well known for its important place in history over the centuries. Aristotle, Archimedes and Hippocrates stand for major scientific, mathematical and medical principles. Democracy, Western philosophy and literature and the Olympic Games originate in this country, and today its archaeological richness, natural beauty (the Aegean and Ionian islands, the mainland) and its friendly and hospitable people make the country an attractive place to visit. However, Greece is also a country where natural disasters occur quite frequently, road traffic accidents cause thousands of people to be injured or lose their life, and child abuse and neglect has immediate and long term mental and physical health effects.

At the same time, Greeks are resilient and overcome adversities. Until now, there are no epidemiological studies on Greek (youth) mental health and studies on trauma and resilience are absent (Southwick, Bonanno, Masten, Panter-Brick, & Yehuda, 2014). On top of the adversities listed above, Greek society is struggling economically which might impact the present and future of the younger population in particular (Kolaitis & Giannakopoulos, 2015). Although the effect of the financial crisis on the mental health of young people is still unknown, it is expected to produce a youth mental health crisis.

Thousands of educated young people have left Greece (the so-called ‘brain drain’) to find work abroad. Increasing numbers of children and their families with complicated psychosocial adversities and sometimes trauma are seen as outpatients or admitted as inpatients. A characteristic and disturbing example is the number of neglected or abused children admitted for child protection to the largest Greek paediatric hospital before they end up in an institution; this number has risen from 81 cases in 2011 to 170 cases in 2014 (Kolaitis & Giannakopoulos, 2015). Studies in adults show a significant association between major depression, suicide mortality and economic hardship (Economou, Madianos, Peppou, Patelakis, & Stefanis, 2013; Kontaxakis et al., 2013).

Greece is not among the top 20 of most publishing countries in the world on trauma or trauma related pathology (Olff & Vermetten, 2013). However, considering its relatively small population of 10,816,286 people (2011) and relative lack of supporting research, Greece has significantly contributed to the scientific literature. During the last 15 years or so, research in Greece has been expanding and researchers have tried to study various populations exposed to different common potentially traumatic situations and events.

Below we present examples of major areas of psychotrauma research in Greece, followed by current research as presented in the Athens conference 19–20 May 2017. The conference was organized by the Greek Society of Child Mental Health and Neuropsychiatry in Athens, on Psychological Trauma in Children and Adolescents. More than 200 professionals and young scientists attended this successful conference which is expected to be developed into an annual international conference. The programme included a welcome by Ntre, president of the organizing society, presentations on trauma across the genders (Olff, 2017), on trauma and PTSD in youth populations (Kolaitis, 2017) and on the impact of trauma and PTSD on physical and mental health or neurobiological outcomes (Agorastos, 2017; Lazaratou, 2017; Pervanidou, Agorastos, Kolaitis, & Chrousos, 2017; Thomadaki, 2017); presentations addressed trauma and psychiatric sequelae in different populations (Belivanaki, Ropi, Kanari, Tsiantis, & Kolaitis, 2017; Charitaki, Pervanidou, Tsiantis, Chrousos, & Kolaitis, 2017; Farmakopoulou, Triantafyllou, & Kolaitis, 2017; Triantafyllou, Ntre, & Kolaitis, 2017) and specific forms of psychotherapy of trauma and PTSD (Kalantzi-Azizi & Anastasiou, 2017; Papanikolopoulos & Prattos-Spongalides, 2017; Syros, 2017).

## 1. Greek mythology and modern war history

Since 2008, the Theater of War (Brooklyn, New York), has presented the works ‘Ajax’ and ‘Philoctetes’ by Sophocles to over 80,000 US military service members, veterans and their families, in American military bases around the world; in these performances and discussions, participants share their own traumatic experiences as part of catharsis through the ancient drama.

The historical course of the modern Greek nation is characterized by a series of ambitious ventures, inevitable catastrophes and rescues by allies with a more or less positive outcome (Kalyvas, 2015). The country has experienced, among others, the Greek-Turkish war (1919–1922) which ended up with the Minor Asia catastrophe, the traumatic occupation by the Axis Powers (1941–1944, with more than 40,000 civilian deaths in Athens from starvation, terrible hardships and a destroyed economy and infrastructure), a brutal civil war (1946–1949) and two huge waves of migration abroad (one-sixth of the population in 1890–1914, one million in 1950–1974) for economic and political reasons (the 1946–1949 civil war and the 1967–1974 period of military junta rule).

## 2. Current research in Greece

### 2.1 Natural disasters

The 1999 Athens earthquake is the most studied natural disaster in Greece in terms of the psychosocial effects on children, adolescents and adults (Giannopoulou et al., 2006; Goenjian et al., 2011; Groom & Soureti, 2004; Kolaitis et al., 2003; Roussos et al., 2005). This was a 5.9 Richter earthquake followed by 4000 aftershocks, had its epicentre in the north-western outskirts of Athens and was the most devastating in last 50 years, as it resulted in 143 deaths, more than 400 injuries and damage to 74,067 households. All studies found high rates of PTSS in the earthquake exposed group 4–6 months following the event compared to indirectly exposed (Giannopoulou et al., 2006) or non-exposed (Kolaitis et al., 2003) groups.

In summer 2007, a series of massive forest fires broke out across Greece. The fires affected western and southern Peloponnese as well as southern Euboea. In total 84 people lost their lives because of the fires, including several fire fighters. A total of 670,000 acres of forest, olive groves and farmland were destroyed in the fires. The fires destroyed 1000 houses and 1100 other buildings, and damaged hundreds more. High levels of PTS, depressive and anxiety symptoms (45, 34 and 32%, respectively) were found four months after the disaster in Lakonia prefecture (Kolaitis et al., 2011).

Some studies focused on the 1986 Kalamata earthquake and its psychosocial effects on adults. High rates of Acute Stress Reaction (ASR) (Soldatos, Paparrigopoulos, Pappa, & Christodoulou, 2006) and protracted ASR (Bergiannaki, Psarros, Varsou, Paparrigopoulos, & Soldatos, 2003) were found; ASR was found to be the only significant predictor for the development of PTSD (Soldatos et al., 2006). According to Papadatos, Nikou and Potamianos (1990), 31.3% of the general population, 11.6% of students and 40.6% of patients exhibited 10 or more symptoms on the Langner scale indicating severe disturbance. Also 50.9% of the whole sample exhibited six or more symptoms indicating serious disturbance. On the Center for Epidemiological Studies Depression Scale (CES-D) scale, 74.6% of the whole sample exhibited 16 or more symptoms (Papadatos et al., 1990).

Triantafyllou presented an outline of disasters, their impact on youth and preliminary findings of a recent study on the 2014 Cephalonia earthquakes comparing them with those of a previous study of the 1999 Athens earthquakes (Triantafyllou et al., 2017).

### 2.2. Child maltreatment

Child abuse has been studied in a large Balkan Epidemiological Study on Child Abuse and Neglect. The Greek data showed that the incidence and prevalence were 47.38 and 76.37% for physical violence, 70.02 and 83.16% for psychological violence, 9.54 and 15.84% for inappropriate sexual experience (including physicalcontact 4.45 and 7.60%), and 26.41 and 37.20% for self-reported subjective feelings of neglect (Petroulaki, Tsirigoti, Zarokosta, & Nikolaidis, 2013). Greece is a country in which corporal punishment is not uncommon as a means of disciplining children; therefore, this piece of data should be treated with great caution and not interpreted necessarily as cases of abuse. In a recent study of undergraduate and postgraduate students of Athens universities, high rates of early traumatic experience were found; the most frequent types of trauma were corporal punishment (89.9%), emotional abuse (67.2%) and sexual abuse (27%) (Antonopoulou, Konstantakopoulos, Τzinieri-Coccosis, & Sinodinou, 2017). Belivanaki’s presentation focused on a previous study on traumatic exposure to violence and PTSD among psychiatric inpatient children and adolescents (Belivanaki et al., 2017).

### 2.3 Road traffic accidents (RTAs)

During the last five years in Greece, 4231 persons lost their lives because of RTAs while more than 58,000 were injured (many of them with lifelong disabilities and handicaps). These are relatively high numbers for the population of Greece of people who may be at risk of acute stress disorder or PTSD and would benefit from psychosocial care. Children involved in traffic accidents may deserve special attention as they also may suffer mental health consequences. Guilt after RTAs for instance has been found to predict acute stress disorder among children (Haag, Zehnder, & Landolt, 2015). Charitaki presented data from a prospective study of PTSD that was performed in hospitalized children and adolescents immediately after a RTA, and one and six months later (Charitaki et al., 2017).

## 3. The refugee crisis

The recent waves of traumatized refugees from Syria and other countries have contributed to the already existing economic crisis in Greece. Since 2015, some 860,000 refugees and migrants without travel documents have entered the country, via the Greek islands, on their way to other European countries. Almost 47,000 of them have remained in Greece (UN Refugee Agency, 2016). Migrant and refugee populations experience traumatic stress on several levels (Hall & Olff, 2016) but may benefit from adequate trauma-focused treatment (e.g. Acarturk et al., 2015). There remain challenges in treatment of refugees that require contextual and culture-sensitive perspectives (Drožđek, 2015). Refugee children may be at risk in particular. Farmakopoulou presented two cases of refugee children in a paediatric hospital (Farmakopoulou et al., 2017) and Kalantzi-Azizi presented a pilot study of an intervention for refugee children (Kalantzi-Azizi & Anastasiou, 2017). The two cases and everyday clinical practice confirm that many young people and their parents have been exposed to various stressors, been traumatized and experience health and mental health problems, such as PTSD and depressive and anxiety symptoms. It is imperative that this population is recognized and helped (Anagnostopoulos, Triantafyllou, Xylouris, Bakatsellos, & Giannakopoulos, 2016).

## 4. Trauma and the need for collaboration

The present state of research on psychotrauma in Greece requires it to be embedded in a community of scientists and practitioners that share the same goals: that of sharing of knowledge and experience about all aspects of psychotraumatology, by fostering research and best practice, building networks and contributing to public policy. Currently the Greek Psychotrauma Society is being established which will hopefully become a member society of the European Society for Traumatic Stress Studies (ESTSS; www.estss.org), fostering international research and improving clinical practice. We totally agree with colleagues that more intense global collaboration is needed in the field of psychotraumatology to be more effective together (Alisic, Jongmans, van Wesel, & Kleber, 2011; Olff, 2016).
